# Small excretory–secretory proteins of *Acanthamoeba castellanii* genotype T4 upregulate the expression of capsule-associated genes via the Rcs two-component system in *Klebsiella pneumoniae*

**DOI:** 10.1186/s13071-025-07123-0

**Published:** 2025-11-19

**Authors:** Yen-Chu Chuang, Hsin-Yu Hsieh, Chih-Ming Tsai, Yu-Jen Wang

**Affiliations:** 1 School of Medicine, China Medical University, Taichung, Taiwan; 2 Department of Nursing, Feng Yuan Hospital, Ministry of Health and Welfare, Taichung, Taiwan; 3 Department of Parasitology, College of Medicine, National Cheng Kung University, Hsinchu, Taiwan; 4 Department of Parasitology, School of Medicine, China Medical University, Taichung, Taiwan; 5 Graduate Institute of Biomedical Sciences, China Medical University, Taichung, Taiwan; 6 Departments of Laboratory Medicine and Internal Medicine, China Medical University Hospital, Taichung, Taiwan

**Keywords:** *Acanthamoeba castellanii*, Hypervirulent *Klebsiella pneumoniae*, Rcs two-component system, Excretory–secretory proteins

## Abstract

**Background:**

Opportunistic pathogens such as *Klebsiella pneumoniae* and *Acanthamoeba castellanii* frequently coexist in environmental water bodies, where their interactions can influence bacterial pathogenicity. Our previous work demonstrated that *A. castellanii* genotype T4 (AcT4) can induce capsule enlargement in *K. pneumoniae*, suggesting that it is a potential environmental source of hypervirulent strains. However, the molecular mechanisms underlying this transformation remain unclear.

**Methods:**

We investigated the effects of AcT4 on the capsule formation and virulence of *K. pneumoniae* using co-culture assays, antibiotic susceptibility testing, lactate dehydrogenase (LDH) cytotoxicity assays, and serum resistance assays. Capsule-associated gene expression (*wzi*, *galF*, *rcsB*, *rmpA*, and *rmpA2*) was analysed via reverse transcription–quantitative polymerase chain reaction (RT-qPCR). Excretory–secretory proteins (ESPs) from AcT4 were separated into >10 kDa and <10 kDa fractions to identify active components. Ion concentrations in amoeba-conditioned media were measured to assess environmental stress factors.

**Results:**

Co-culture with AcT4 significantly increased *K. pneumoniae* cytotoxicity toward A549 cells and enhanced serum resistance without altering antibiotic susceptibility. RT-qPCR revealed significant upregulation of *rcsB*, *galF*, and *wzi* in the induced strains, while the expression of the virulence plasmid genes *rmpA/rmpA2* was absent. Amoeba-conditioned media altered ion distributions, notably increasing iron levels, and ESP fractionation revealed <10 kDa molecules as the primary drivers of capsule enlargement and *wzi* upregulation.

**Conclusions:**

Small (<10 kDa) ESPs from AcT4 can induce capsule-associated gene expression in *K. pneumoniae* via activation of the Rcs two-component system, independent of virulence plasmids. These findings reveal a novel contact-independent mechanism by which environmental protozoa may contribute to the emergence of hypervirulent *K. pneumoniae*, highlighting potential public health risks from aquatic environments.

**Graphical Abstract:**

**Figure:**
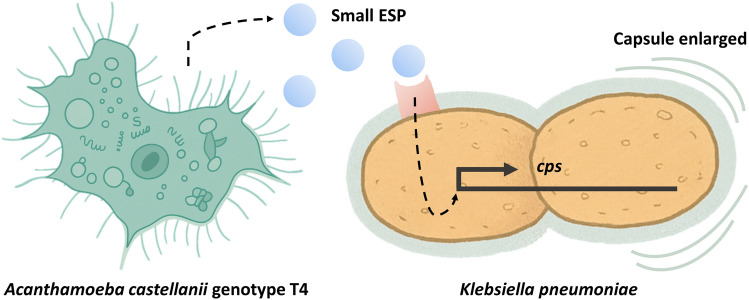

## Background

Many opportunistic pathogens are dormant in the environment before invading the human body [1, 2]. Bacteria and protozoa coexist in the environment for extended periods, leading to physiological changes in these microorganisms. The primary characteristics of protozoa are their phagocytic ability [3] and the release of extracellular proteins [4]. Several previous studies have confirmed that physiological changes in bacteria can help them evade predation and molecular influences by protozoa. For instance, after being phagocytosed by free-living amoebae, intracellular *Legionella* spp., *Chlamydophila pneumoniae*, and *Mycobacterium avium* can become resistant to protozoan digestion [5]. This allows protozoa to provide a favourable environment for these pathogens to evade external environmental pressures. Owing to the extracellular molecular influences of protozoa, *Acanthamoeba castellanii* and *Colpoda maupasi* have also been shown to affect population dynamics within biofilm communities, including those of *Klebsiella pneumoniae*, *Pseudomonas fluorescens*, and *Staphylococcus epidermidis* [6]. Notably, in addition to the interactions between protozoa and bacteria inducing various mechanisms that allow bacteria to persist in the environment, these physiological changes also alter the risk of opportunistic infections in humans.

Chlorine is among the most commonly used disinfectants to ensure the safety of drinking water [7]. The dissolution of chlorine in water generates hypochlorous acid or hypochlorite ions, which destroy bacterial structures through oxidation reactions [8]. However, protozoa, especially those capable of forming cysts, are often overlooked in tap water, despite their presence in environments where they can thrive. After interacting with various bacterial species, *A. castellanii*, *Tetrahymena pyriformis*, and *Cyclidium* spp. have been shown to increase bacterial resistance to chlorine in water by 50% [9]. Such protozoan–bacteria interactions occur even in treated tap water, posing a significant threat to human water safety. *Pseudomonas aeruginosa*, which is also a common environmental commensal, relies on colonization and biofilm formation as key factors during opportunistic infections. Notably, the presence of *A. castellanii* can increase *P. aeruginosa* accumulation in areas of cell monolayer leakage and promote biofilm formation [10]. Such effects of *A. castellanii* on bacteria increase the likelihood of opportunistic infections by *P. aeruginosa*. Therefore, it is imperative to continue exploring the interactions between various environmental protozoa and bacteria.

*Klebsiella pneumoniae* is a common gram-negative bacterium found in environmental water bodies. Previous sampling studies have shown that this bacterium is present in wastewater discharged from factories [11], downstream river water [12], irrigation lakes [13], and even domestic tap water [14]. Recently, a new hypervirulent strain of *K. pneumoniae* (hvKp) has been reported to cause severe clinical effects and exhibit transnational spread [15]. However, the exact conditions leading to the formation of hypervirulent strains remain unclear. Interestingly, our previous research demonstrated that *A. castellanii* genotype T4 (AcT4), a common free-living eukaryotic microorganism in aquatic environments, can interact with *K. pneumoniae* and induce capsule enlargement in the bacteria [16]. These findings offer a new avenue for exploring the sources of hvKp. The capsule enlargement phenomenon in *K. pneumoniae* can persist in subsequent generations of bacterial populations and can be induced by *A. castellanii* across several serotypes. The interaction between two waterborne commensals is not surprising, but such changes could pose significant public health concerns.

In this study, we continued to explore the impact of AcT4 on the capsule of *K. pneumoniae*. The antibiotic resistance and immune resistance of the bacteria were used to elucidate the potential harm to humans following their interaction. Gene expression analysis and protein fraction experiments were conducted to clarify the pathways involved in protozoa-induced capsule biosynthesis. Through this research, a clearer understanding of the interaction between these two commensals was achieved.

## Methods

### Cultivation of *Acanthamoeba* protozoa

The T4-genotype *Acanthamoeba* strain, which was isolated from soil, was identified as the *A. castellanii* Neff strain (ATCC-30010) and was purchased from the American Type Culture Collection (ATCC; Manassas, VA, USA). The ATCC-30010 strain was originally isolated from soil in Pacific Grove, California, and the cultivation and maintenance methods were the same as those we used previously [17]. Briefly, *A. castellanii* cells were cultured at 28 °C in cell culture flasks supplemented with proteose peptone–yeast extract–glucose medium (20 g proteose peptone, 18 g glucose, 2 g yeast extract, 1 g sodium citrate dihydrate, 0.98 g MgSO_4_, 0.34 g KH_2_PO_4_, 0.188 g Na_2_HPO_4_ × 7H_2_O, 0.02 g Fe(NH_4_)_2_(SO_4_)_2_ × 6H_2_O in 1 L of ddH_2_O [pH 6.5]). The cells were then washed and resuspended three times with Page’s modified Neff amoeba saline (1.2 g NaCl, 0.04 g MgSO_4_-7H_2_O, 0.03 g CaCl_2_, 1.42 g Na_2_HPO_4_, and 1.36 g KH_2_PO_4_ in 1 L ddH_2_O).

### Cultivation of *Klebsiella pneumoniae*

The *K. pneumoniae* isolate was cultured at 35 °C on Luria–Bertani (LB) agar (solidified with 1.5% [w/v] agar) or in LB broth. The isolate, identified as K-81, was previously obtained from the corneal surface of a hospitalized patient, and its use was approved by the Institutional Review Board (IRB) of National Cheng Kung University Hospital (Protocol No. B-ER-109-108; approval date: 8 June 2020) [18].

### Antibiotic susceptibility testing

*Klebsiella pneumoniae* co-cultured with AcT4 was inoculated onto LB agar plates and incubated overnight at 35 °C. Using a sterile inoculation loop, four to five colonies were transferred into 4–5 ml of tryptic soy broth (TSB) in test tubes. The cultures were incubated at 35 °C until they reached a McFarland standard of 0.5. The bacterial suspension was then evenly spread in three directions on Mueller‒Hinton agar using a sterile cotton swab. After waiting for 3–5 min, the antibiotic discs were placed onto the agar surface. The plates were incubated overnight at 35 °C, and the inhibition zones were measured the following day.

### Cytotoxicity assay

Human lung carcinoma A549 cell monolayers were grown, and the medium was replaced with fresh cell culture medium (Dulbecco’s modified Eagle medium [DMEM] + 10% foetal bovine serum [FBS] + 1% Pen-Strep) 24 h before infection. AcT4-induced or un-induced bacteria were extracted by centrifugation (5 min at 1200×*g*) and resuspended in DMEM without supplements, such that the final optical density at 600 nm (OD)_600_ was 0.1. The medium of monolayer A549 cells was replaced with 250 μl of DMEM without supplements. Next, 250 μl of DMEM supplemented with bacteria was added to the culture medium of monolayer A549 cells. Infected plates were then incubated in a humidified incubator for 8 h at 37 °C/5% CO_2_. The cytotoxicity of the cells was determined by measuring lactate dehydrogenase (LDH) release into the supernatants (Cytotoxicity Detection Kit, Roche). Using a Multiskan SkyHigh Microplate Spectrophotometer (Thermo Scientific), the absorbance of the product generated from the reaction with LDH was measured at 492 nm. On the cell monolayers, Triton-X 100 was used to kill all of the cells (positive control). Untreated cells, on the other hand, functioned as the negative control. The following formula was used to convert the absorbance to cytotoxicity: (sample value − negative control value)/(positive control value − negative control value) × 100 = cytotoxicity % [19].

### Serum resistance assay

*Klebsiella pneumoniae* strains were co-cultured overnight with AcT4 to induce transformation. After incubation, the bacterial cultures were harvested, and the bacterial concentration was adjusted to an OD_600_ of 0.2. The adjusted suspensions were then treated with pre-warmed normal pooled human serum (Sigma-Aldrich) for 2, 4, and 6 h at 37 °C, with OD measurements taken at each time point. Following the density measurements, the samples were plated on Mueller‒Hinton agar and incubated overnight at 35 °C. The next day, images of the colony-forming units (CFU) were captured to assess the serum resistance of the bacteria.

### India ink staining

A mixture of equal parts culture and India ink (Sigma-Aldrich, St. Louis, MO, USA) was prepared on a glass slide, followed by the application of a coverslip. Microscopic examination was conducted using an Olympus BX51 microscope at 1000× magnification.

### Quantification of capsule-related genes

The expression of capsule-associated genes in *K. pneumoniae* was assessed using reverse transcription–quantitative polymerase chain reaction (RT-qPCR) with the *wzi* primer set *wzi*_for (5′-GTGCCGCGAGCGCTTTCTATCTTGGTATTCC-3′) and *wzi*_rev (5′-GAGAGCCACTGGTTCCAGAACTTCACCGC-3′) at 55 °C, the *galF* primer set *galF*-F (5′-CAAAGGCAATTCCAAAGGAG-3′) and *galF*-R (5′-TGCGTCACCAGAACAATCTC-3′) at 57 °C, the *rcsB* primer set *rcsB-*F (5′-CAAATACGGCGACGGGAT-3′) and *galF-*R (5′-CAGCGAGACGGAAGAGAGGT-3′) at 54 °C, the *rmpA* primer set RT-17 (5′-TCAATAGCAATTAAGCACAAAAGAA-3′) and RT-18 (5′-TTGTACCCTCCCCATTTCC-3′) at 53 °C, and the *rmpA2* primer set *rmpA2*-F (5′-AAATCATTACCCACAACTAACAAAAA-3′) and *rmpA2*-R (5′-TTAGACGGCTTTTTAATTCATGG-3′) at 53 °C. Total RNA was extracted from *K. pneumoniae* using the Direct-zol™ RNA MiniPrep kit (Zymo Research, CA) with DNase I treatment to remove any residual genomic DNA. A 400-ng aliquot of the purified RNA was reverse transcribed and then amplified via PCR using SYBR green dye (Invitrogen) on an ABI StepOnePlus™ thermocycler (Applied Biosystems, Foster City, CA, USA). The threshold cycle (Ct) for each gene was determined and compared against the Ct of the *16S* ribosomal RNA gene from the corresponding complementary DNA (cDNA) sample. Relative RNA expression levels were then calculated on the basis of the Ct values.

### Separation of AcT4 excretory–secretory proteins

The conditioned medium, which was collected after overnight culture of AcT4 cells, was processed using a centrifugal filter unit (Amicon Ultra-4; Millipore Corporation, Bedford, MA, USA). The medium was centrifuged at 3000×*g* for 30 min, resulting in two distinct fractions: Fraction 1 (>10 kDa) and Fraction 2 (<10 kDa). These separated fractions were then incubated with *K. pneumoniae* for 24 h.

### Analysis of external ions

Pages modified with Neff’s amoeba saline (PAS) buffer, either cultured with or without 3 × 10^5^AcT4 cells, were collected after 1 and 5 days of incubation. The PAS samples were centrifuged at 540×*g* for 3 min to eliminate any debris. The supernatants were then analysed using a COBAS 8000 automated analyser (Roche). The results are based on three independent experiments, with statistical significance determined using Student’s *t*-test.

### Statistical analysis

All experiments were performed in three independent biological replicates, unless otherwise specified. Each biological replicate was measured in technical triplicate, and the average value was used for analysis. The data are presented as the mean ± standard deviation (SD). Statistical analyses were performed using a two-tailed Student *t*-test to compare two groups. All data are presented as mean ± SD from three independent biological replicates, each measured in technical triplicate. A *P*-value < 0.05 was considered statistically significant. The number of replicates (*n*) and the statistical test used are indicated in each figure legend.

## Results

### AcT4 interaction enhances hypervirulence and cytotoxicity in *K. pneumoniae* without altering antibiotic susceptibility

**Fig. 1:**
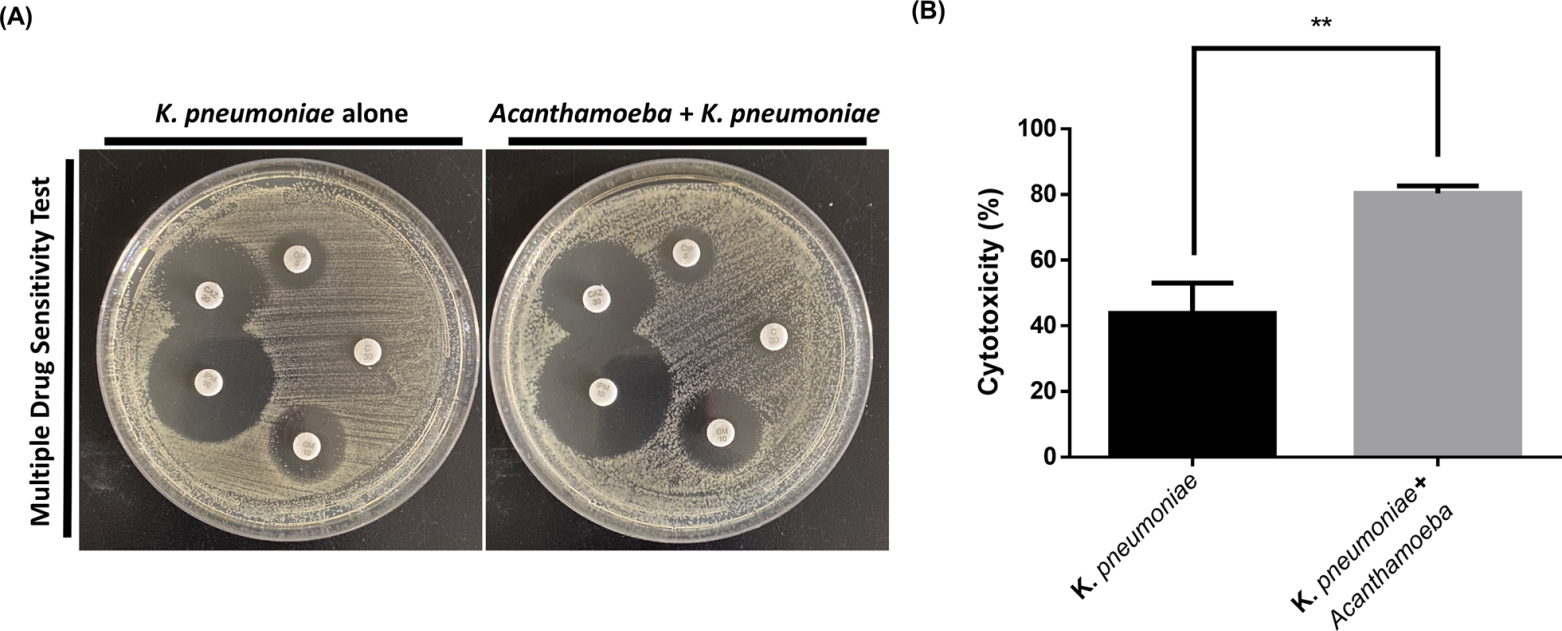
Antibiotic susceptibility and cytotoxicity tests of AcT4-stimulated *K. pneumoniae*. **A** *Klebsiella pneumoniae* strains that were either co-cultured overnight with AcT4 or cultured alone were adjusted to a McFarland standard of 0.5 and evenly spread on Mueller‒Hinton agar. Following another overnight incubation, the inhibition zones of the respective antibiotics were measured. *GM* gentamicin, *C* chloramphenicol, *CIP* ciprofloxacin, *CAZ* ceftazidime, *IPM* imipenem. **B** *Klebsiella pneumoniae* cytotoxicity to the non-small cell lung cancer cell line A549. Cytotoxicity was measured by the detection of lactate dehydrogenase (LDH) concentration in the medium after 8 h of co-culture. Data represent *n* = 3 independent biological replicates, each measured in technical triplicate. Statistical significance was determined using a two-tailed Student *t*-test (**P < 0.01)

In previous studies, we demonstrated that AcT4 could induce capsule enlargement in *K. pneumoniae* during co-culture. However, to gain a more comprehensive understanding of the impact of protozoal interactions on *K. pneumoniae* in the context of infection and treatment, we conducted antibiotic susceptibility tests to assess the changes in drug sensitivity induced by AcT4. Given that *K. pneumoniae* possesses multiple antibiotic resistance mechanisms, including enzymatic degradation and efflux pump systems [20], we utilized a range of antibiotics for the experiments. These included the aminoglycoside gentamicin (GM), the commonly used combination treatment agent chloramphenicol, the fluoroquinolone ciprofloxacin (CIP), the cephalosporin ceftazidime (CAZ), and the carbapenem imipenem (IPM). After overnight culture, *K. pneumoniae* that was co-cultured with AcT4 and those cultured alone were plated and exposed to antibiotic discs. On the basis of the measurements of the inhibition zones, there was no significant difference in the inhibition zones between AcT4-induced *K. pneumoniae* and *K. pneumoniae* cultured alone (Fig. 1A). These findings suggest that the ability of AcT4 to induce transformation in *K. pneumoniae* may not influence its antibiotic susceptibility. Although most hvKp strains possess a thick capsule and exhibit hypermucoviscosity, the hypervirulence of *K. pneumoniae* is not always associated with its mucoviscosity [21]. Therefore, we proceeded to conduct cytotoxicity assays on *K. pneumoniae* following its interaction with AcT4.

Using the release of LDH from lysed mammalian cells as an indicator, we observed that, compared with *K. pneumoniae* that was not co-cultured with AcT4, AcT4-induced *K. pneumoniae* caused significantly higher levels of cell death in A549 cells after 8 h of infection (Student’s t-test, two-tailed *P* < 0.01) (Fig. 1B). This suggests that the AcT4-induced strain is a hypermucoviscous hvKp.

### Enhanced serum resistance and survival of *K. pneumoniae* induced by AcT4

**Fig. 2:**
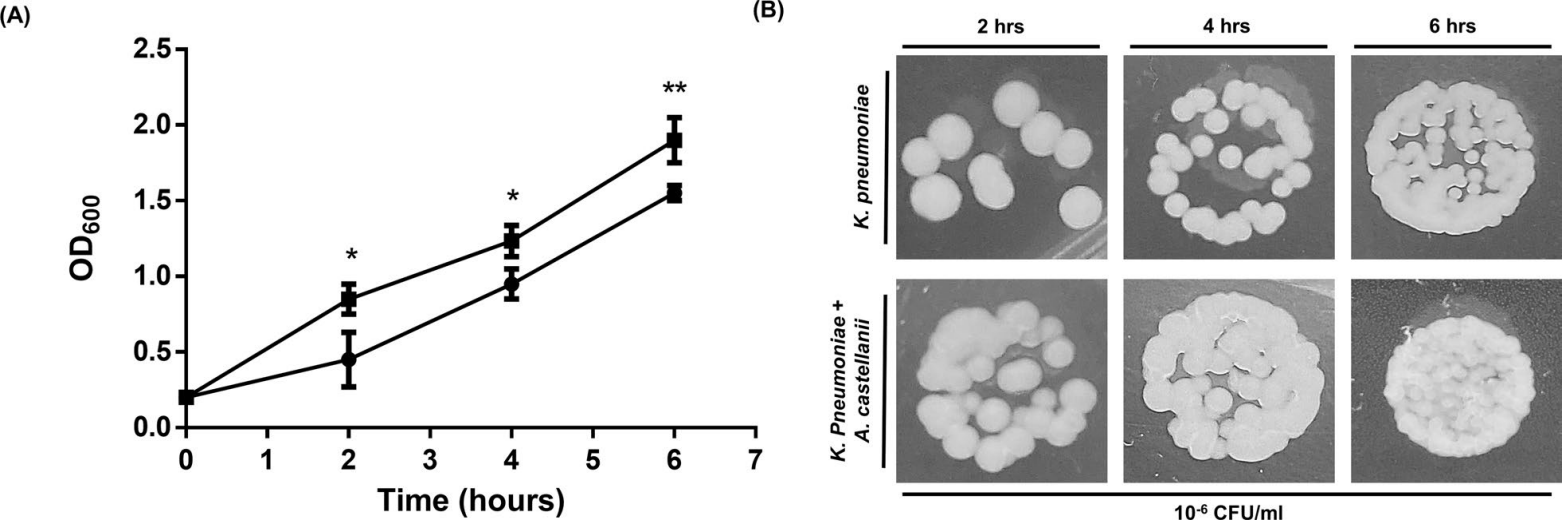
Serum resistance assay of *K. pneumoniae* following co-cultivation with AcT4. **A** Bacterial optical density at 600 nm of co-cultured (■) and non-co-cultured (●) *K. pneumoniae* with AcT4 after post-incubation with normal human serum for 2, 4, and 6 h. The *y*-axis displays the OD_600_ measurements of treated *K. pneumoniae*, and the significance of the comparison was calculated using Student’s *t* test. **B** The image shows the CFU counts at 10^−6^ CFU/ml for *K. pneumoniae* treated with normal human serum for 2, 4, and 6 h. Data represent *n* = 3 independent biological replicates performed with pooled human serum, each measured in triplicate. Statistical significance was assessed using a two-tailed Student *t*-test (*P < 0.05 or **P < 0.01 as indicated)

*Klebsiella pneumoniae* is an opportunistic pathogen capable of causing a variety of infectious syndromes, with sepsis being particularly common in neonates [22]. Therefore, we next used normal human serum to assess the immune resistance of AcT4-induced *K. pneumoniae*. To assess the resistance of induced *K. pneumoniae* to inhibition by human serum, we first co-cultured classical *K. pneumoniae* (cKp) with AcT4 overnight. The following day, the induced *K. pneumoniae* was harvested. The adjusted *K. pneumoniae* suspensions at an OD_600_ of 0.2 were then incubated with pre-warmed normal human serum for 2, 4, or 6 h. At each serum incubation time point, bacterial growth was measured by determining the OD_600_. The measured values for *K. pneumoniae* cultured alone versus AcT4-induced *K. pneumoniae* were as follows: 2 h: 0.45 vs. 0.85 (Student’s *t*-test, two-tailed *P* < 0.05); 4 h: 0.95 vs. 1.23 (Student’s *t*-test, two-tailed *P* < 0.05); and 6 h: 1.55 vs. 1.90 (Student’s *t*-test, two-tailed *P* < 0.01) (Fig. 2A). These findings indicate that although serum immune factors may not have sustained effects in vitro, compared with *K. pneumoniae* cultured alone, AcT4-induced *K. pneumoniae* exhibited significantly greater resistance under serum inhibition at each time point. Additionally, for each serum-incubated *K. pneumoniae* sample, we performed serial dilutions and cultured them overnight at 35 °C to assess colony formation. Similarly, on the basis of the CFU images, the results were consistent with the OD measurements, indicating that AcT4-induced *K. pneumoniae* formed more colonies (Fig. 2B). These findings further indicate that induced *K. pneumoniae* is more resistant to serum immune factors. Moreover, the effect induced by AcT4 may contribute to an increased survival rate of *K. pneumoniae* under the suppression of human immunity.

### Microenvironmental stress induced by AcT4 promotes Rcs-mediated capsule enlargement in *K. pneumoniae* via ion redistribution

**Fig. 3:**
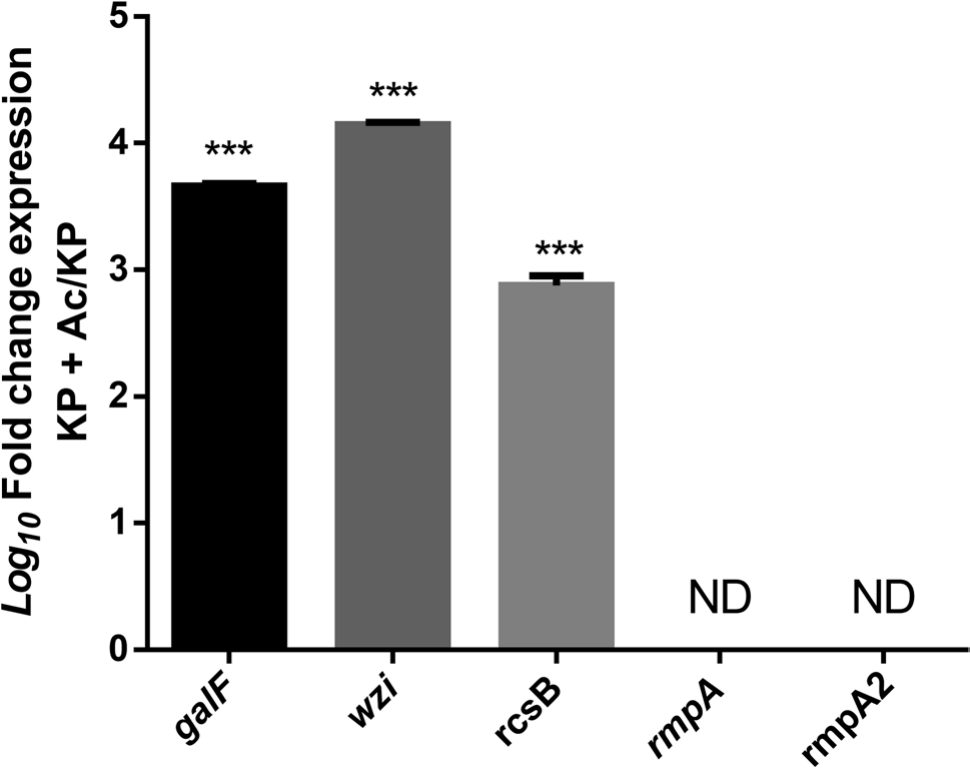
Relative expression of *K. pneumoniae* capsule-associated genes. The expression levels of capsule-associated genes in *K. pneumoniae*, including *galF* and *wzi* within the *cps* region, *rcsB* of the two-component system, and *rmpA* and *rmpA2* from a large virulence plasmid, were compared. Gene expression was normalized to that of the *16S* ribosomal RNA gene. The abbreviations used are KP for *K. pneumoniae*, Ac for *A. castellanii* genotype T4, and ND for non-detected *K. pneumoniae*. The gene expression data are presented as *n* = 3 independent biological replicates, each measured in technical triplicate. Differences were analysed using a two-tailed Student *t*-test (***P < 0.001)

The Rcs two-component system on the bacterial cell surface and the virulence plasmid within the cell are currently considered the primary factors contributing to the hypervirulence and hypermucoviscosity of *K. pneumoniae* [21]. However, the precise pathways through which AcT4 induces hvKp remain unclear. Therefore, we proceeded to analyse the expression of Rcs two-component system genes, virulence plasmid genes, and capsule biosynthesis genes through qPCR. According to the statistical results of gene relative abundance, the expression level of the Rcs two-component system gene *rcsB* was significantly upregulated in AcT4-induced *K. pneumoniae* (Student’s *t*-test, two-tailed *P* < 0.001). Additionally, the expression of the downstream capsule biosynthesis genes *galF* and *wzi* was significantly upregulated (Student’s *t*-test, two-tailed *P* < 0.001). However, virulence plasmid genes were not detected in either AcT4-induced or non-induced *K. pneumoniae* (Fig. 3).

**Fig. 4:**
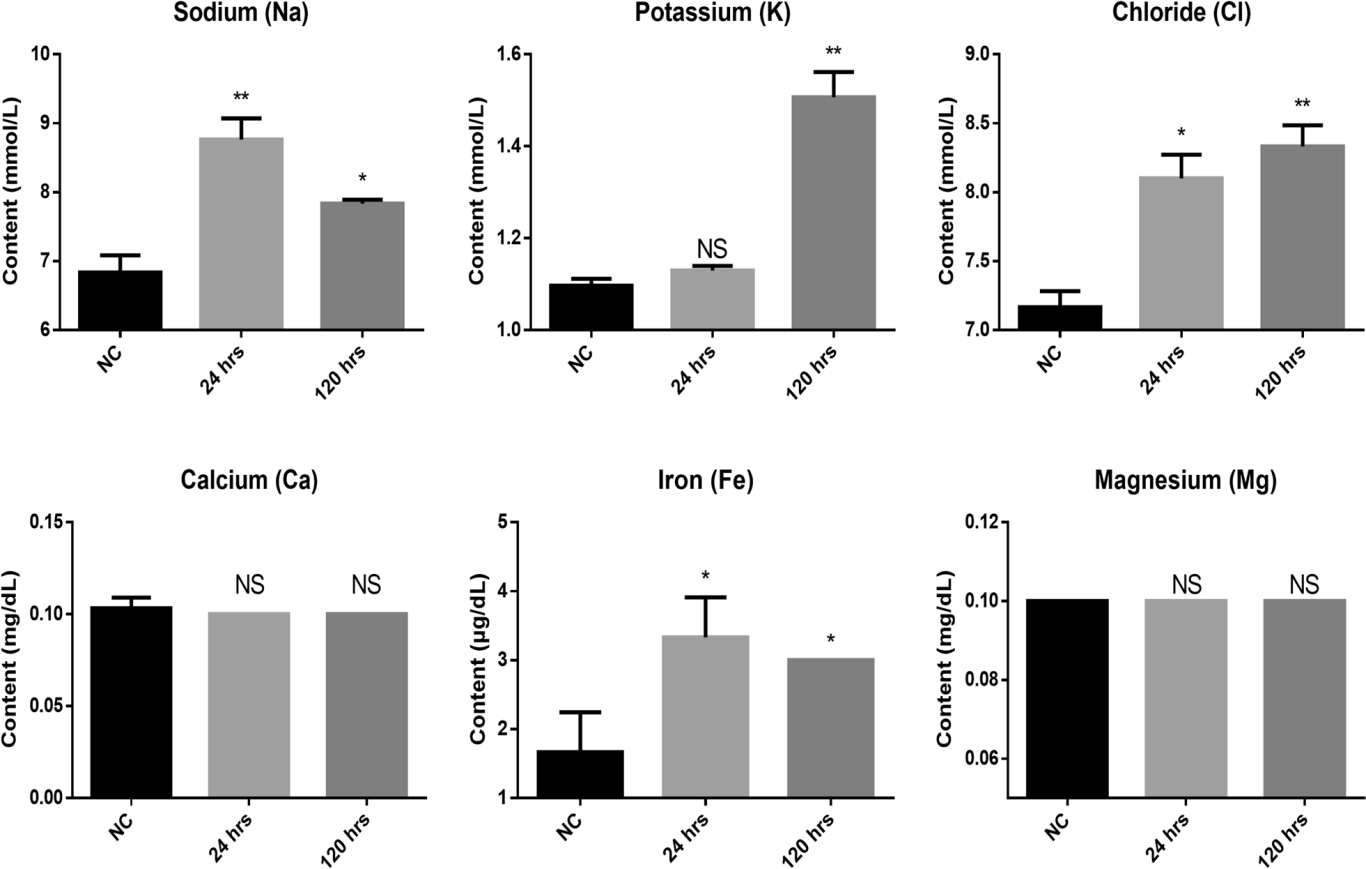
Analysis of extracellular metal ions in AcT4-conditioned medium. The conditioned medium from AcT4 cultures was collected after 24 and 120 h of incubation. This medium was then analysed for concentrations of sodium, potassium, chloride, calcium, iron, and magnesium using an automated biochemical analyser. A negative control (NC) was included in the analysis. Data represent *n* = 3 independent biological replicates, each measured in technical duplicates. Statistical significance between groups was assessed using a two-tailed Student *t*-test (*P < 0.05 or **P < 0.01 as indicated). NS indicates no significant difference (*P* > 0.05)

The Rcs two-component system is present in many members of the *Enterobacteriaceae* family of gram-negative bacteria [23]. Various environmental stresses, including increased osmolarity and redox stress [24, 25], have been shown to activate the Rcs system. Therefore, we extracted the conditioned medium of AcT4 and analysed the changes in ion concentrations using a chemical analyser. When the blank medium was compared with the AcT4-conditioned medium after 24 and 120 h of culture, significant changes were observed in the concentrations of sodium, potassium, and chloride ions, which were related to osmolarity (Student’s *t*-test, two-tailed, 120 h: sodium: *P* < 0.05; potassium: *P* < 0.01; chloride: *P* < 0.01). Additionally, among the metal ions commonly found in microenvironments, iron ions were significantly increased (Student’s *t*-test, two-tailed, 24 and 120 h: *P* < 0.05) (Fig. 4). These findings suggest that AcT4 alters the ion distribution in its microenvironment, thereby inducing microenvironmental stress that activates the Rcs system on the surface of *K. pneumoniae* cells lacking a virulence plasmid. This activation leads to the upregulation of downstream *cps* locus gene expression, ultimately resulting in the enlargement of the *K. pneumoniae* capsule.

### Small-molecule proteins in AcT4 excretory–secretory proteins drive *K. pneumoniae* capsule biosynthesis

**Fig. 5:**
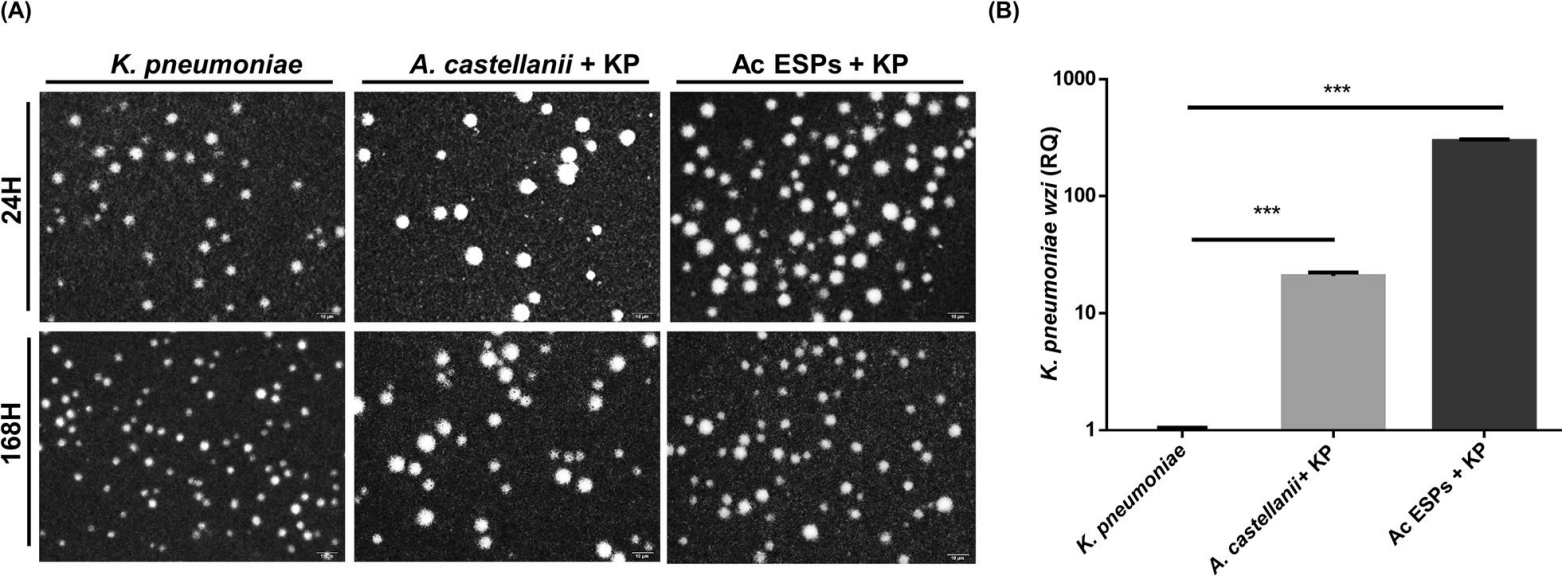
Influence of AcT4 excretory–secretory proteins on capsule formation and *wzi* gene expression in *K. pneumoniae*. **A** India ink staining of *K. pneumoniae* under different conditions after 24 h and 168 h of incubation. The three conditions included *K. pneumoniae* alone, *K. pneumoniae* co-cultured with AcT4, and *K. pneumoniae* treated with excretory–secretory proteins (ESPs) from AcT4. The images highlight the differences in capsule formation and morphology of *K. pneumoniae* under each condition over time. Representative images from *n* = 3 independent biological replicates are shown. **B** Quantitative analysis of *K. pneumoniae wzi* gene expression following treatment with AcT4 ESPs. Gene expression levels were measured using qRT‒PCR, and the results are presented as relative quantification (RQ) compared with the control. Data represent *n* = 3 independent biological replicates, each measured in technical triplicate. Statistical significance was determined using a two-tailed Student *t*-test (***P < 0.001)

*Acanthamoeba castellanii* is known to excrete and secrete various proteins and extracellular vesicles (EVs) into the microenvironment [26, 27]. These excretory–secretory proteins (ESPs) are thought to cause host cell damage and modulate environmental stress. Therefore, to further investigate whether the ion changes in the microenvironment are due to AcT4 ESPs and whether these proteins can induce *K. pneumoniae* capsule biosynthesis in a contact-independent manner, we next extracted the AcT4-conditioned medium and compared its effects with those of AcT4 cells in stimulating *K. pneumoniae*. Using India ink staining and relative expression analysis of the *wzi* gene by qPCR, we found that after *K. pneumoniae* was incubated with AcT4 ESPs for 24 and 168 h, the results were consistent with those previously observed when *K. pneumoniae* was induced by AcT4 cells. Specifically, *K. pneumoniae* exhibited an enlarged capsule under India ink staining (Fig. 5A), and the expression of the capsule biosynthesis gene *wzi* was significantly upregulated (Student’s *t*-test, two-tailed *P* < 0.001) (Fig. 5B).

**Fig. 6:**
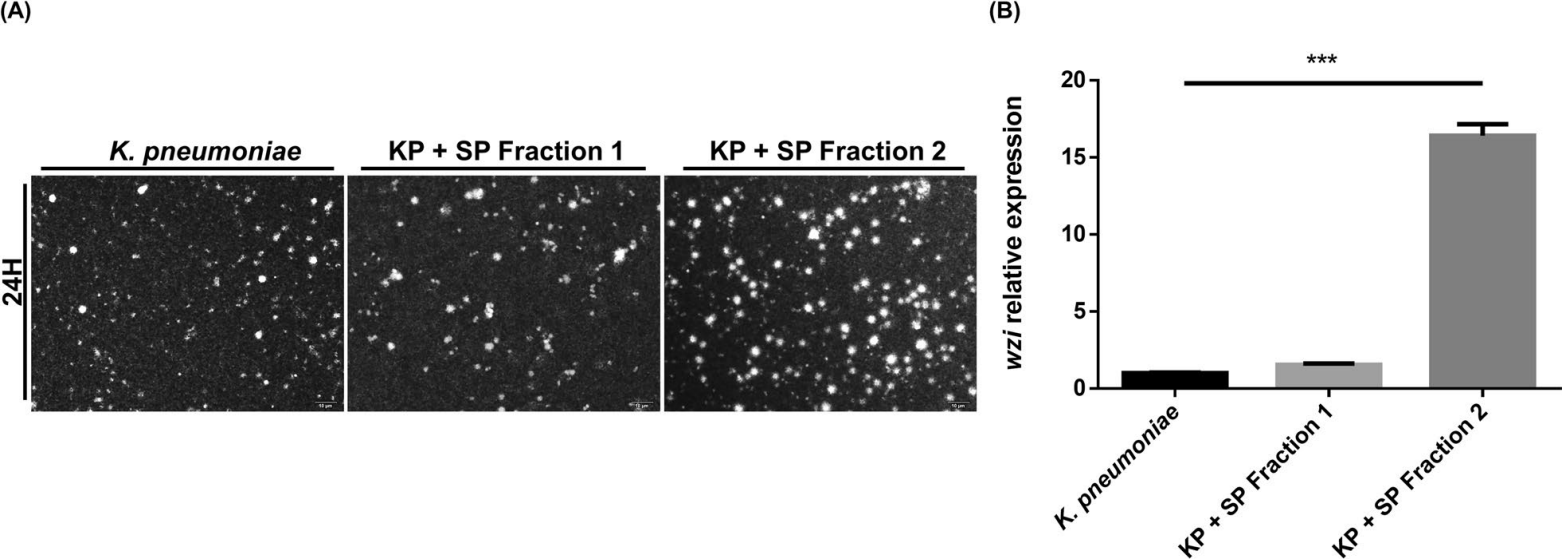
Effect of AcT4 ESP fractions on the *K. pneumonia* capsule. **A** India ink staining of *K. pneumoniae* after 24 h of incubation under three different conditions: *K. pneumoniae* alone, *K. pneumoniae* treated with ESP Fraction 1 (>10 kDa) from AcT4, and *K. pneumoniae* treated with ESP Fraction 2 (<10 kDa) from AcT4. The images show differences in capsule formation and bacterial morphology under these conditions. Representative images from *n* = 3 independent biological replicates are shown. **B** Quantitative analysis of *wzi* gene expression in *K. pneumoniae* following treatment with ESP Fraction 1 (>10 kDa) and ESP Fraction 2 (<10 kDa). Gene expression levels were measured using RT‒qPCR, and the results are presented as the relative quantification (RQ) compared with those of *K. pneumoniae* without ESP treatment. Data represent *n* = 3 independent biological replicates, each measured in technical triplicate. Statistical significance was determined using a two-tailed Student *t*-test (***P < 0.001)

However, ESPs in protozoa are diverse, with a wide range of molecular weights, from enzymes larger than 300 kDa to EVs smaller than 10 kDa [28, 29]. Therefore, we separated the AcT4-conditioned medium into two fractions using ultrafiltration with a 10 kDa cut-off—a high-molecular-weight fraction (Fraction 1) and a low-molecular-weight fraction (Fraction 2)—which were then added to *K. pneumoniae* for further analysis. Using the same staining and gene expression analysis methods, we found that only Fraction 2 was capable of inducing *K. pneumoniae* capsule enlargement (Fig. 6A) and upregulating the expression of the *wzi* gene (Fig. 6B). These findings suggest that the small-molecule proteins within AcT4 ESPs are the primary factors responsible for the increased capsule biosynthesis in *K. pneumoniae*.

## Discussion

Most opportunistic pathogens persist in the environment for an extended period rather than directly infecting humans. As a result, these microorganisms are not only pathogens but also act as commensals in the environment [2]. However, from a practical standpoint, these opportunistic pathogens exhibit differences in terms of their virulence and defence mechanisms. Consequently, virulence traits may have evolved to enhance their environmental adaptability [30]. Among these factors, protozoa, which coexist in the environment as both commensals and pathogens, are considered among the elements that can induce changes in the virulence of these microorganisms. The interactions between protists and bacteria significantly influence bacterial virulence and infectivity. For example, *Legionella pneumophila* becomes more resistant to environmental stressors and antibiotics after passing through amoebae, enhancing its ability to infect human hosts [31]. Similarly, the virulence of *Vibrio cholerae* increases after it is packaged into expelled food vacuoles by ciliates, preconditioning the bacteria to survive harsh conditions and improving their ability to colonize hosts [32]. These interactions act as “training grounds” where bacteria can develop and refine virulence traits, leading to increased pathogenicity even after their passage through protists.

However, there are also cases in which bacterial virulence changes without bacteria being phagocytosed. For example, *P. aeruginosa* exhibits phenotypes similar to those of strains isolated from cystic fibrosis patients, including increased antibiotic resistance and persistence, after co-cultivation with the social amoeba *Dictyostelium discoideum* [33]. Another example is *Salmonella enterica*, where the presence of ciliates or other protists can increase the expression of acid resistance genes, thereby increasing their survival and virulence in the gut environment [34]. These examples highlight the fact that even extracellular interactions with protists can drive significant changes in bacterial virulence, underscoring the complex role that protists play in bacterial evolution and pathogenicity. Interestingly, although many bacteria can be phagocytosed and maintained as endosymbionts within *Acanthamoeba*, previous work has shown that *K. pneumoniae* does not survive intracellularly in *A. castellanii*. Electron microscopy and GM protection assays confirmed that the bacteria remained extracellular during co-culture, in contrast to their ability to be internalized by human macrophages (THP-1 cells) [35]. These findings suggest that structural features such as the thick polysaccharide capsule of *K. pneumoniae* likely provide resistance against amoebic engulfment, thereby preventing phagocytosis and endosymbiosis.

We previously reported interactions between AcT4 and multiple *K. pneumoniae* serotypes (K1, K2, K5, K20, and K81) [16], demonstrating that amoeba-induced capsule enlargement and hypermucoviscosity occur across diverse strains. These findings suggest that the effect is not limited to a single isolate but may be broadly applicable. In the present study, we selected isolate K-81 as a representative strain to perform detailed mechanistic assays. Since both organisms are commensals in aquatic environments—including rivers [36, 37], saltwater [38, 39], and wetlands [40, 41]—gaining insight into this discovery is crucial. *Klebsiella pneumoniae* is a common and challenging source of nosocomial infections in many countries [42], with the clinical issues it causes stemming primarily from antibiotic-resistant and highly virulent strains. However, since a research team in Taiwan first reported on hvKp, many subsequent studies have focused primarily on its cellular physiology and genetic aspects [43]. Research on its origins, such as whether hvKp represents a distinct strain from cKp or whether hvKp has evolved from cKp, remains relatively limited. Therefore, our research findings offer a potential explanation for this important issue. Moreover, on the basis of the literature related to environmental sampling, the occurrence of such interactions between these microorganisms is not unexpected.

hvKp is typically characterized by hypermucoviscosity and capsule overexpression, which are regulated by the Rcs system and/or rmpA plasmid genes. It has been demonstrated that *rcsB* mutants in strains CG43 and KPPR1S exhibit reduced *cps* expression, leading to a decrease in uronic acid content [44]. However, through isogenic gene deletion and complementation experiments, Hsu and colleagues demonstrated that *K. pneumoniae* can regulate *cps* expression via the expression of *rmpA* genes from plasmids [45]. Therefore, whether the upstream trigger responsible for hypervirulence in hvKp is primarily the Rcs two-component system or the *rmpA*/*rmpA2* plasmid remains unclear.

On the basis of our discovery that AcT4 can induce the formation of hvKp, we conducted an expression analysis of genes involved in the possible pathways that could induce cps expression in this study. Interestingly, the *K. pneumoniae* strain we originally used for interaction with AcT4 apparently lacked a virulence plasmid. However, genes related to the two-component system, particularly *rcsB*, were found to have increased expression. Additionally, the expression of the capsule-related genes *galF* and *wzi* was also increased. Here, we propose an alternative possibility for the induction of hvKp. In natural environments, even cKp strains lacking virulence plasmids may still transform into hvKp because of stimulation from surrounding AcT4. Additionally, we provide evidence that the presence of *rmpA*/*rmpA2* plasmids is not an absolute requirement for the formation of hvKp.

Iron acquisition systems are crucial for the virulence of *K. pneumoniae*, although excess iron can be detrimental [46]. The transcriptional regulator *Fur* is involved in the processes of iron uptake and metabolism and has also been reported to modulate the Rcs system [47]. In parallel, the iron–sulfur cluster regulator IscR directly activates capsule biosynthesis genes in an Fe-S–dependent manner, linking iron availability to capsule production and hypervirulence [48]. In our study, AcT4 increased extracellular iron levels, and conditioned medium alone was sufficient to induce hvKp formation. These findings suggest that iron may contribute to capsule biosynthesis through Fe-S–dependent regulation, while <10 kDa ESPs secreted by amoebae likely trigger Rcs activation through envelope/osmotic stress. Together, these parallel pathways converge on the upregulation of capsule-associated genes. Classic work has also shown that Na^+^, K^+^, and divalent cations modulate encapsulation independent of growth [49], and more recent studies revealed that the capsule behaves as an “ion sponge” responsive to osmotic stress [50]. Thus, alterations in ionic composition and amoebal ESPs may act synergistically to drive capsule remodelling and hvKp formation.

Nevertheless, the exact identity of these <10 kDa ESPs remains undefined, which represents a limitation of the present study. Future work will aim to characterize the active components through proteomic approaches such as liquid chromatography–tandem mass spectrometry (LC‒MS/MS), followed by validation with recombinant candidate proteins to confirm their role in capsule induction. Notably, our findings are based solely on in vitro assays. The ecological and clinical implications of amoeba-derived ESPs should therefore be interpreted with caution, and future validation using animal infection models and environmental surveillance will be necessary to confirm their in vivo relevance.

## Conclusions

This study demonstrated that small (<10 kDa) ESPs from AcT4 can induce capsule-associated gene expression in *K. pneumoniae* via activation of the Rcs two-component system, independent of virulence plasmids. These findings reveal a novel contact-independent mechanism by which environmental protozoa may drive hypervirulence in bacterial pathogens, highlighting the potential risk of aquatic environments as reservoirs for clinically significant strains. Our future work will focus on identifying and characterizing these small-molecule ESPs to better understand their role in microbial interactions and to inform strategies for preventing and controlling hvKp infections.

## Data Availability

Data supporting the main conclusions of this study are included in the manuscript.

## Abbreviations

AcT4: *Acanthamoeba castellanii* genotype T4
cKp: Classical *K. pneumonia*
CAZ: Ceftazidime
CIP: Ciprofloxacin
ESPs: Excretory–secretory proteins
GM: Gentamicin
hvKp: Hypervirulent *Klebsiella pneumonia*
IPM: Imipenem
LDH: Lactate dehydrogenase
